# A Smart Walker for People with Both Visual and Mobility Impairment

**DOI:** 10.3390/s21103488

**Published:** 2021-05-17

**Authors:** Nafisa Mostofa, Christopher Feltner, Kelly Fullin, Jonathan Guilbe, Sharare Zehtabian, Salih Safa Bacanlı, Ladislau Bölöni, Damla Turgut

**Affiliations:** 1 Department of Computer Science, University of Central Florida, Orlando, FL 32816, USA; nafisa.mostofa@knights.ucf.edu (N.M.); chris.feltner@knights.ucf.edu (C.F.); jguilbe@knights.ucf.edu (J.G.); sharare.zehtabian@knights.ucf.edu (S.Z.); bacanli@knights.ucf.edu (S.S.B.); Ladislau.Boloni@ucf.edu (L.B.); 2 Department of Computer Science, Ashland University, Ashland, OH 44805, USA; kellyfullin@gmail.com

**Keywords:** smart walker, obstacle detection, aging, rehabilitation

## Abstract

In recent years, significant work has been done in technological enhancements for mobility aids (smart walkers). However, most of this work does not cover the millions of people who have *both* mobility and visual impairments. In this paper, we design and study four different configurations of smart walkers that are specifically targeted to the needs of this population. We investigated different sensing technologies (ultrasound-based, infrared depth cameras and RGB cameras with advanced computer vision processing), software configurations, and user interface modalities (haptic and audio signal based). Our experiments show that there are several engineering choices that can be used in the design of such assistive devices. Furthermore, we found that a holistic evaluation of the end-to-end performance of the systems is necessary, as the quality of the user interface often has a larger impact on the overall performance than increases in the sensing accuracy beyond a certain point.

## 1. Introduction

While a significant amount of research and industry interest targets mobility aids at the elderly and disabled, these efforts are often not applicable to people who have specific comorbidities. A particularly widespread group of such patients have both visual and mobility impairments. For instance, according to the World Health Organization, there are an estimated 1.3 billion people globally living with some form of visual impairment [1]. As this population ages, they will also require mobility assistance at least at the rate of the people with a healthy vision, estimated to be about 16% for individuals 65 years of age or older [2]. A significant challenge, however, is that the assistive technologies developed for people with normal eyesight often cannot be used by people with visual impairments. This is partially due to the fact that the user interfaces often rely on visual feedback. Furthermore, people with visual impairment already need some type of assistive technology to navigate their environment—the use of two distinct devices would lead to an unacceptable cognitive overload.

The research described in this paper focuses on devices that simultaneously help people with their visual and mobility impairments, and at the same time also use user interfaces which are appropriate to the capabilities of the user and do not lead to cognitive overload. As there has been very little work done on devices with this particular combination of capabilities (some examples include [3,4]), it is not clear what type of path planning and obstacle detection technologies are appropriate in these settings (ultrasound, vision, infrared and or structured/light technologies). It is also uncertain what type of user interaction is the least distracting (sound, haptic or high-contrast visual) and what the content and frequency of the communication with the user should be. In conclusion, one of our objectives was the exploration of the design space: we investigated a variety of obstacle detection and user interaction technologies, and evaluated them under various scenarios. The four obstacle detection techniques we used are (a) ultrasonic distance sensors; (b) 2D observable light camera input processed with state-of-the-art deep learning-based computer vision algorithms; (c) depth images from an RGB-D camera with minimal post-processing; and (d) the processing of a 3D point cloud acquired from an RGB-D camera. For the user interface, we investigated the use of audible and haptic signals.

Beyond the investigation of the technologies involved, we need to keep in mind that the output of our work needs to be an assistive device that can be deployed and used by people who have mobility and visual impairments. We implemented the technological solutions in the form of a smart walker, and we also took into consideration several practical design requirements. The walkers need to be affordable, they should preferably be autonomous, and do not require a network connection or cloud computation. Furthermore, the user interface is very important, as it needs to convey information about obstacles and proposed avoidance strategies without distracting the user.

The contributions of this paper are as follows:

- We described the designs of several smart walker configurations that can use various technologies for obstacle detection and user interaction.
- We described four different approaches for obstacle detection, based on different sensing techniques (ultrasound, RGB cameras, and RGB-D depth cameras). Correspondingly, we described the appropriate processing techniques that can transform the sensor output to a signal identifying the detected obstacles.
- We validated the proposed object detection techniques in a series of real-world experiments.
- We studied the user interaction techniques based on audio versus haptic notifications.

The remainder of the paper is organized as follows. In Section 2, we review the related work. In Section 3, we described the proposed approaches in detail. The evaluations of the proposed approaches through various configurations are given in Section 4 and we conclude in Section 5.

## 2. Related Work

One of the earliest implementations for a smart walker and smart cane for elderly users with impaired mobility were the PAMM designs implemented at MIT in the late 1990s (Dubowsky et al. [5]). These devices were essentially small mobile robots augmented with handles. They used a sonar array for obstacle detection, an upward pointing camera for localization using ceiling mounted markers and a force/torque sensor mounted on the handle for user control. While limited by the technology available at the time, these designs remained influential, and outlined the research directions which are now being pursued by many researchers.

MacNamara and Lacey [3] proposed a wheeled walker (rollator) targeted towards aged people who also have visual impairments. Similarly to our work, this system was designed to detect obstacles in the path of the user and communicate their presence using two techniques: audio feedback and a feedback that uses motors to align the wheels in an obstacle-free direction.

This work led to an early commercial implementation in the form of the motorized rollator Guido by Haptica [6]. In addition to a more polished commercial design, this system specifically focused on older blind people. It used sonar and laser ranging devices to avoid obstacles and a SLAM algorithm to build a map of its environment, allowing it to guide the user to a predetermined destination. One of the challenges faced by Guido as a commercial product launched in the early 2000s was the high cost of devices such as LIDARs.

Zehtabian et al. [7] described an IoT-augmented four-legged walker, which uses sensors to track and visualize the weight distribution over its legs during use. This facilitates proper walker usage for rehabilitation and assists physicians in checking their patients’ rehabilitation progress.

In a follow-up paper, Khodadadeh et al. [8] processes the walker’s data stream using a deep neural network-based classifier. This allows the detection of unsafe usage that could hinder a patient’s rehabilitation.

Paulo et al. [9] implemented ISR-AIWALKER, a robotic walker using computer vision as the primary human–machine interface modality. This contrasts with previous approaches that primarily relied on force sensing. The walker was also augmented with multimodal sensing capabilities that allow it to analyze and classify the gait of the user. In follow-up work, Garrote et al. [4] augmented the ISR-AIWALKER with robot-assisted navigation targeted towards users who lack a dexterous upper limb or have visual impairments. The walker uses reinforcement learning algorithms to learn a behavior that fuses user intent and the environmental sensing of the obstacles. Whenever obstacles are detected, the system adds corrections to the movement in order to avoid collisions.

Kashyap et al. [10] developed a self-driven smart walker by augmenting the rear wheels of a rollator with motors. The user interacts with the rollator using voice commands, such as “go to (room name)”. The authors evaluated several off-the-shelf, LIDAR-based simultaneous localization and mapping (SLAM) implementations for mapping and navigation. The system also has a fall detection algorithm that prevents the rollator from moving away if the user falls.

Bhatlawande et al. [11] proposed a system where ultrasonic sensors are attached to a belt and a pair of glasses worn by the user. The system detects and identifies obstacles and indicates an obstacle-free path using audio feedback.

Orita et al. [12] implemented a device that augments the white cane used by visually impaired people with a Microsoft Kinect camera connected to a laptop and uninterruptible power supply in a backpack. Information about the lack of obstacles was communicated to the user through vibration feedback. A small experimental study using blindfolded subjects had shown that the device helped users navigate an indoor environment.

Pham et al. [13] devised a system to provide a blind user feedback about drops, objects, walls, and other potential obstacles in the environment. The system relies on a Kinect sensor worn by the user, with the output processed by a laptop computer in a backpack. Feedback to the user is provided using a Tongue Display Unit, a sensory substitution device.

Panteleris and Argyros [14] investigated the challenges of vision-based SLAM arising in the use of the c-Walker [15], a smart rollator with a Kinect sensor as an RGB-D camera. Rollator users normally move in environments with many other people around, thus the SLAM algorithm must consider a large number of independently moving objects.

Viegas et al. [16] described a system which allows a four-legged smart walker to collect data about the load the users put on each walker leg using load cells and the relative position of the user to the walker using a LIDAR. This information is transmitted using Bluetooth to an external device and can be used to guide the users in the correct use of the device and prevent dangerous situations.

Ramadhan [17] described a wrist-wearable system that allows visually impaired persons to navigate public places and seek assistance if needed. The system contains a suite of sensors, haptic interaction modules as well as a GPS module and has the ability to communicate over cellular networks.

Kim and Cho [18] performed a user study about the challenges encountered by the users of several types of commercial smart canes with obstacle detection capabilities, and compared them with the traditional white canes. The output of the customer interviews was used to advance design guidelines for improved smart canes.

Feltner et al. [19] and Mostofa et al. [20] designed walkers targeted at visually impaired people. These works describe the early versions of the walker configurations described in the next section.

## 3. Proposed Approaches

The most widely used assistive devices for mobility are the cane, four wheeled rollators, and four legged walkers. These devices are simple and intuitive for most people and, by physical construction, they support and stabilize the users. In order to extend the benefits of these devices to people who are both mobility *and* visually impaired, they need to be augmented with additional capabilities. A relatively wide number of choices exist with regards to the type of sensing and processing capabilities that can be deployed. To explore this design space, we started from a standard four-wheeled rollator with a basket and seat. We implemented four different augmentation configurations across a range of sensor types, processing hardware and software, and user interface techniques.

### 3.1. User Interaction

Assistive devices in general, and devices for visually impaired users in particular, have special user interface (UI) requirements. Graphical user interfaces, the most widely used techniques to convey information to the user, are not applicable. UIs for assistive devices must be robust to environmental noise, not require significant cognitive effort from the user and reduce the chance of misunderstood signals. Given the capabilities of the augmented rollator, there are two distinct messages that the UI must convey. The *obstacle detected* message warns the users that they would hit an obstacle if they continue on the current trajectory. A signal of increased urgency can be used to convey the proximity of the obstacle. The *navigational guidance* message provides a recommendation to the user to turn towards the left or right in order to avoid the obstacle.

For our rollator, we chose to implement and compare two UI methods: a voice-based user interface that conveys the information through spoken messages, and a haptic feedback that is enacted through coin vibration motors attached to the handles of the walker. Both modalities can convey both the obstacles detected and the navigational guidance messages, as well as their various gradations. In the case of the voice interface, this is conveyed through the content and tone of the voice. For the haptic interface, the intensity of the vibration corresponds to the proximity of the obstacle, while the vibration in the left or right handle conveys the direction of the recommended turn.

### 3.2. (A) Ultrasonic Sensors

In this configuration, we removed the basket of the walker and attached nine HC-SR04 [21] ultrasonic sonar distance sensors to the lower front crossbar of the walker. We used a Raspberry Pi 3b+ device to drive the sensors, as well as collect and interpret the results.

**Figure 1:**
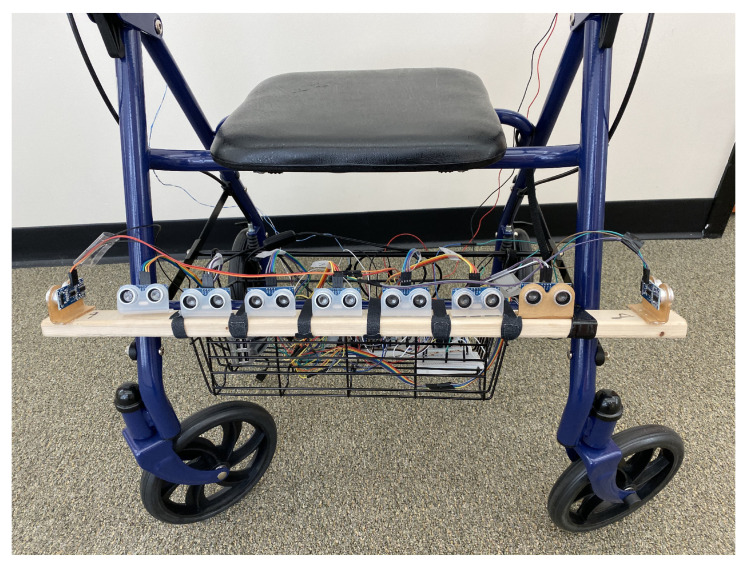
The rollator configured with nine HC-SR04 ultrasonic sensors. Seven sensors are facing forward to capture obstacles in a wide area in front of the walker. To facilitate navigational guidance, two sensors (one on the left and the other on the right) are capturing obstacles.

The HC-SR04 sensor operates as follows. A ultrasonic sound wave, above the frequency of human hearing, is generated as a trigger. If there is an object in the sensor’s path, the sound wave bounces back to the sensor as an echo and is captured by the sensor. By measuring the time taken by sound to travel to the obstacle and back, we can calculate the distance to the obstacle. In practice, this sensor is limited to a viewing angle of 14 degrees and can operate to a distance of up to 400 cm–s with an accuracy of 3 mm. In order to cover the range of obstacles of interest to the user of the walker, we attached seven of these sensors across the width of the walker. They were also angled slightly towards the floor in order to detect obstacles immediately in the front of the walker. In addition, to detect obstacles to the left and right of the walker, we attached one outward angled sensor to each side of the walker (see Figure 1). One technical challenge we encountered was that the sound waves from the multiple sensors interfered with each other, which is possible for one sensor to detect a delayed echo from a wave started by a different sensor. To avoid this, in our implementation the sensors perform their detection sequentially.

The outcome of the detection step is an array of distance values corresponding to the directions covered by the individual sensors. The first use of these data is the obstacle detection message: the user will be warned if there is an obstacle closer than 200 cm–s in the direction of movement. This message is triggered if any of the central sensors detect an obstacle in this range. In addition to this, the arrangement of the sensors also allows us to recommend the user a navigational guidance for the avoidance of the obstacle. For instance, if there are obstacles detected in the front and to the right of the walker, the system will recommend the user to move towards the left.

### 3.3. (B) RGB Camera with Deep Learning-Based Computer Vision Algorithms

In recent years, the significant reduction in the cost of video cameras, together with the advances of deep learning-based computer vision algorithms made it possible to detect obstacles based solely on video information, without using a dedicated distance or depth sensors. To investigate the feasibility of such an approach, in this configuration, we mounted a forward-facing Logitech C270HD webcam on the top crossbar of the walker. We used deep learning-based computer vision algorithms to process the video stream. The algorithms required the full-featured version of the Tensorflow library, exceeding the capabilities of a Raspberry Pi device. In our experiments, we used a laptop computer placed on the seating area of the walker.

**Figure 2:**
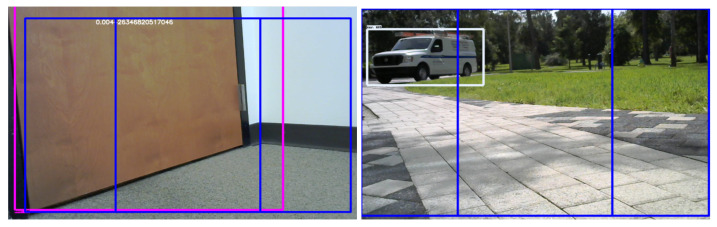
Object detection of door and car with TensorFlow.

Recent research on deep learning-based object detection systems created a number of approaches that can detect objects of specific types in images in real time. Some of these approaches include R-CNN, Fast R-CNN, Faster R-CNN, YOLO and others. While the training of such systems requires non-trivial computational capabilities, many pre-trained neural networks are available in the public domain. For instance, the Object Detection API of the TensorFlow library provides a simple programmatic interface to a choice of several different pre-trained networks. In our experiments, we used a pre-trained network with the Faster R-CNN region proposal network [22] with the Inception Resnet V4 [23] model trained on the Open Images data set [24]. By applying this object detector to the video frames captured through the camera driver of OpenCV, we obtained a collection of bounding boxes on the image, together with the tentative label and a confidence value. While the pre-trained network handles and detects a wider variety of objects, we only retain objects detected with the labels relevant to our application, such as doors, cars and chairs (see Figure 2).

Many objects of interest to the user have relatively uniform sizes. Thus, for a given camera with a fixed focal length, the size of a bounding box in the image can allow us to approximate the distance to the object. For instance, if a chair occupies approximately half of the field of view of a camera with the 45° view angle, the distance to the chair will be approximately 2–3 ft. Note that this approximation critically depends on the correct identification of the object—a car with a similar size bounding box would be much farther away. This approach, while limited in absolute precision, in practice allows us to develop a software implementation that provides sufficient accuracy for the purposes of obstacle detection and navigational messages.

We identify the central region of the visual area, which is where the walker is currently heading. However, comparing the bounding box of the obstacle to this region, we can identify whether the obstacle is in this region, and also if it is on the left or right side of the region. This allows us to generate the appropriate navigational messages.

### 3.4. (C) Depth Camera with Direct Processing of the Depth Image

**Figure 3:**
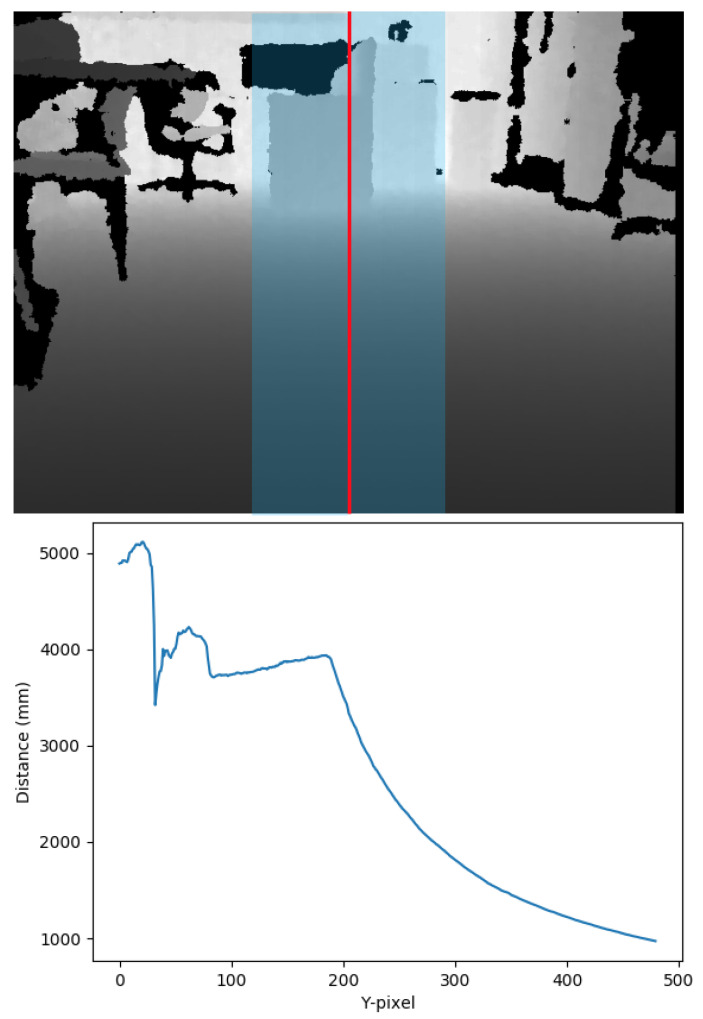
Top: The depth component of an image recorded by a Kinect camera in configuration (C). Darker colors show closer points, with black pixels representing points for which depth information is not available. The blue central column is the area processed for obstacle detection. Bottom: The one-dimensional array extracted from the depth map. Pixel 500 refers to the bottom of the image. The smooth increase in distance from 500 to 200 shows an approximately 4 m free area in front of the walker, with a drop starting after that.

In this configuration, we mounted a Microsoft Kinect RGB-D depth camera to the lower front crossbar of the walker. As with the ultrasonic sensors in configuration (A), the Kinect was angled towards the floor. The device was connected to a Raspberry Pi and powered through a dedicated 12 V DC rechargeable battery. Figure 3—top shows the depth component of a captured image, with darker colors representing closer distances. The areas in black are locations where the camera was unable to determine the location of the point.

In order to implement the functionality required by the walker while relying only on the limited computational capabilities of a Raspberry Pi, we chose to implement an algorithm based on an idea from Ortigosa et al. [25]. We started from the central vertical stripe of the depth image. To reduce the noise and limit the data to be processed, we performed a pre-processing step by calculating the average of the values on the center stripe, skipping the black pixels for which no value was available. This created a one-dimensional array of the size of the height of the image (see Figure 3—bottom). If there is no obstacle in front of the walker, the pixels at the bottom will have the smallest distance values, gradually and smoothly increasing to the top of the image. Obstacles will be indicated by a sudden increase in the slope of this array. On the other hand, a negative slope indicates a drop in the elevation such as the beginning of a staircase or a street curb.

The advantage of this approach is that it only requires an averaging of a central area, followed by an iteration over a one-dimensional array while tracking the slope. This computational load is well within the reach of most IoT devices. A disadvantage of the approach is that by focusing only on the central stripe, it cannot take into consideration the available space to the left and right. Thus, a system using this algorithm can only perform obstacle detection, not navigational guidance.

### 3.5. (D) Depth Camera with Point Cloud Model and Processing

**Figure 4:**
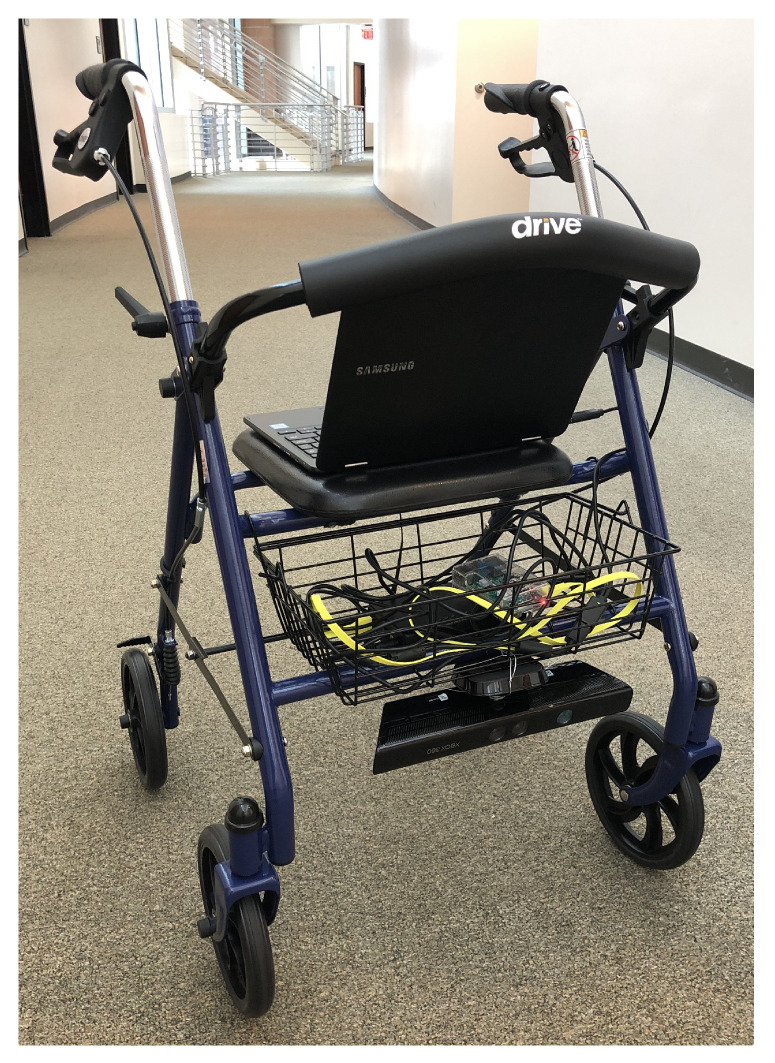
The smart walker in configuration D.

With the Kinect mounted as in case (C), for this configuration we used more complex algorithms that build a point cloud of the scene as an intermediate step. These algorithms required more computational power than available in the Raspberry Pi 3, thus, as in case (B), we used a laptop computer to process the input and to power the Kinect device. Figure 4 shows the rollator in this configuration.

We are extracting a point cloud from the Kinect device, and processing it through an approach similar to that of Pham et al. [13]. In order to focus on the processing of the data relevant to the user of the walker, we performed a series of pre-processing steps using the Point Cloud Library [26]. We removed the points from the point cloud that were not immediately relevant to the user of the walker, as well as points that lie outside the Kinect’s range of accuracy. Second, we noticed that the initially captured point cloud contained hundreds of thousands of points. The user of the walker does not need such a detailed *spatial* resolution for obstacle avoidance. However, the time needed to process this information would reduce the *temporal* resolution of the obstacle detector, introducing unacceptable delays into the obstacle notification and navigation guidance. To solve this problem, we down-sampled the point cloud using a voxel grid filter to a spatial resolution of 1 cm.

The next step is to extract a model of the floor in the point cloud which allows us to interpret other points as belonging to obstacles. We use the random sampling and consensus (RANSAC) algorithm to determine the coefficients of a large horizontal plane within the point cloud, with the inlier points being considered as part of the floor. If no such plane could be found, this means that either the walker is at the edge of a sudden drop (such as a staircase) or that a large, close obstacle obstructed the majority of the camera view. In both cases, the user is warned about an immediate obstacle at close range.

**Figure 5:**
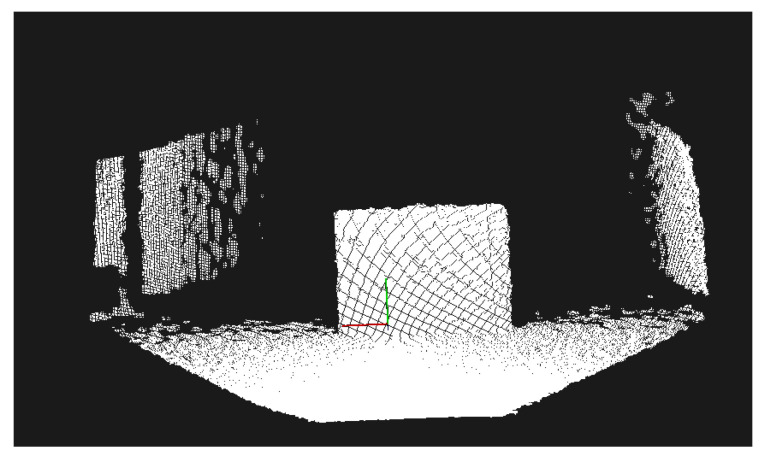

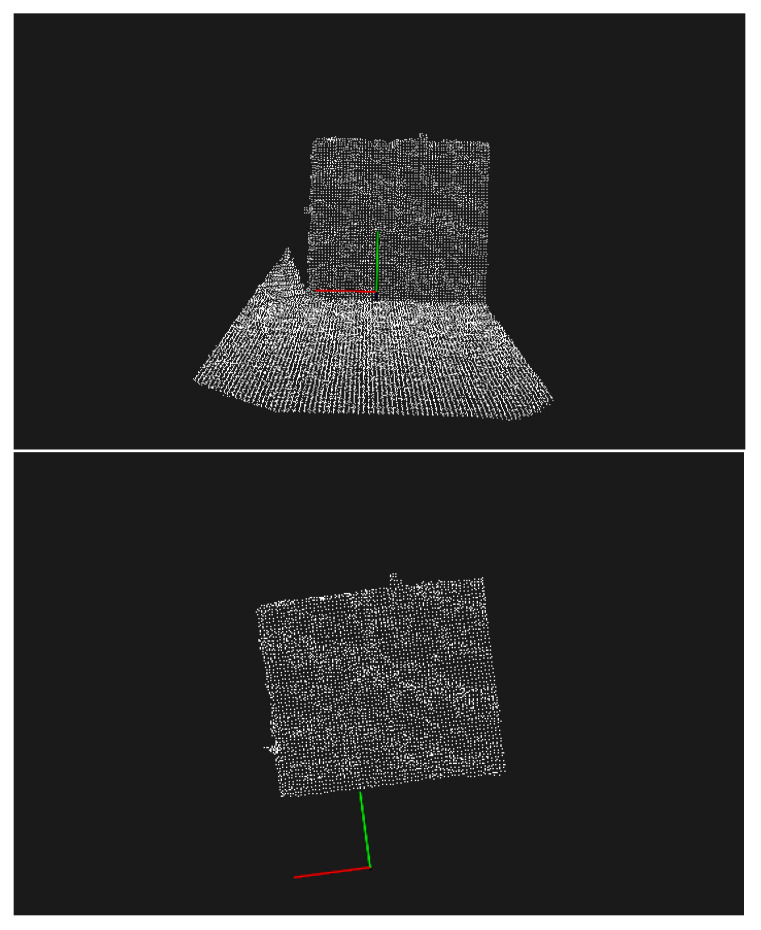
The stages of processing the point cloud: (top) the high-resolution point cloud extracted from the Kinect sensor; (middle) the point cloud after the elimination of non-relevant points and downsampling; and (bottom) the points of an obstacle, after the floor plane was identified and removed from the image using RANSAC.

After the plane of the floor was extracted, the outlier points were considered to be part of the obstacles. We used the techniques described by Li et al. [27] to iterate through all outlier points and determine the distance from the obstacles. The stages of processing the point cloud are shown in Figure 5.

## 4. Results

There is no single criteria that can be used to compare the various configurations of the smart walker. Obviously, devices with higher computational power and more capable sensors should yield better performance when measured in localization accuracy. For instance, an RGB-D camera that returns both an image and a depth value for every pixel will inevitably yield a high-quality model of the environment on which complex path planning algorithms can be applied. However, a sensor of this complexity has both a higher cost and more costly processing requirements.

Sensors with significantly less capabilities, such as the ultrasonic sensors that return only a single numerical value for a depth (configuration (A)), or sensors that collect only an image (configuration (B)), will yield a walker with less capabilities in an absolute sense. However, such a walker can still provide useful services to the user, and be better in terms of its dimensions of cost, robustness and reliability compared to walkers with more complex sensors (such as configurations (C) and (D)).

Thus, we are going to evaluate our designs in two different ways. Objective tests for obstacle detection measure the performance of the sensor paired with a specific processing algorithm. These tests do not involve the participation of a human user.

End-to-end usability tests measure the utility provided by a walker to the user for obstacle avoidance and navigation. Such tests holistically consider the entire system, including sensor performance, processing latency, the quality, and types of feedback given to the user.

The objective of these experiments was to investigate the capabilities of the augmented walker. These tests consider a human user performing specific navigation tasks with the configured walker. As these experimental systems do not yet meet the criteria of safety for human subjects research with elderly and disabled people, in these experiments we used healthy volunteers, from 20 to 30 years old, from the research group. The subjects were instructed to put some of their weight on the walker to model mobility impairment and were blindfolded to model visual impairment.

### 4.1. Obstacle Detection: Configuration (A) versus (B)

In this set of experiments, we compared the two configurations that do not have an RGB-D sensor ((A) and (B)) in their ability to detect and estimate the distance to an obstacle placed in front of the walker.

For the experiments with the (A) and (B) configurations, we chose a set of obstacles to experiment with based on the following considerations. We included both indoor and outdoor obstacles, as these sensor types can function in both environments (this is not possible for (C) and (D)). We also included both obstacles that raise above the level of the floor, and obstacles that represent a drop (such as the stairs, curb and swimming pool). Finally, we added some obstacles that test the ability of the computer vision system in configuration (B) to identify obstacles that cannot be distinguished by a simple distance sensor like in configuration (A). For instance, the computer vision system can distinguish between a door (which can be opened, thus treated differently by the user) from a wall.

What we are interested in here is whether these more limited sensors are able to capture a variety of obstacles that a typical user might encounter. Table 1 shows the results of a series of experiments we performed with a variety of obstacles in a household environment. The ground truth have been obtained through direct measurement from the sensor to the closest point of the obstacle. Some of the conclusions we can draw from these experiments are as follows. Both configurations (A) (ultrasonic sensor) and (B) (camera processed through computer vision) were able to detect all the obstacles in these experiments, and returned the correct “no obstacle” answer in the empty hallway. The ultrasonic sensor, which is an active sensor specialized on the distance measurement, obtained the sub-centimeter accuracy, an operational parameter of this sensor type. As expected, the values obtained from the processing of the camera image were less accurate. The camera, as a passive image sensor, does not directly capture any depth information, as all values are inferred only from relative image sizes. Nevertheless, we conclude that the accuracy of both sensors was sufficient for making a decision about whether the user should be notified of the obstacle or not. As a note, with regards to the quality of this notification, the computer vision system was able to identify the type of obstacle (e.g., person, wall or car), while the depth sensor can only detect the distance, albeit at a higher accuracy.

### 4.2. Obstacle Detection: Configuration (C) versus (D)

In this series of experiments, we compared the two configurations of the walker that use the same Kinect RGB-D camera as the sensor. As discussed in the previous section, the difference between these configurations is the processing algorithms: (C) uses a simple central stripe averaging technique that can be implemented on a Raspberry Pi 3 device, while (D) uses a more complex approach based on creating and processing a point cloud.

A general observation with the use of the Kinect sensor is that due to the fact that it uses an infrared emitter and sensor, it does not work in bright sunshine, when the infrared rays from the sun confuse the measurements. Configurations (A) and (B) do not suffer from this problem. For the experiments with (C) and (D) configurations, the nature of the sensor did not allow for outdoor experiments, which restricted the use of obstacles in the indoor setups. At the same time, the low-resolution point cloud representations are not suitable for certain types of obstacle classifications such as between a wall and a door. On the other hand, the 3D representations allow us to distinguish between obstacles that represent a drop or a raise in the floor. To verify that our algorithms can identify these situations, we added experiments to test for this, looking at the bottom of a stairwell and a drop in the floor level.

The measurements for these obstacle types are shown in Table 2. We found that for these measurements, the approach (D) slightly under-estimated the distance to the obstacles, while (C) slightly overestimated them. However, for all cases, the obstacles and drops were detected correctly, and the errors were small enough not to affect in any measurable way the experience of the user.

We concluded from this experiment that if the only goal was the detection of the obstacles in front of the walker, the much simpler algorithm using configuration (C) is sufficient. However, we note that (C) ignores the obstacles outside of the center stripe, and thus it cannot provide navigational guidance.

### 4.3. Blindfolded Navigation (Configuration A versus B)

As devices need to be deployed to the user, the most useful evaluation is that of measuring the way in which they impact the user’s daily routine. We are less interested, for instance, in the high precision measurement of the distance to an obstacle, than the fact that the obstacle has been detected, and the user had successfully avoided the collision and navigated to their destination with the help of the walker. To evaluate the performance of the augmented walkers along this dimension, we performed a series of experiments that tested the navigational guidance of the walkers for users with severe visual impairment.

In these environments, we considered three setups which involved the same walker frame, but with different configurations and ways of interaction with the user. BASELINE involved the walker without any of the augmentations, serving only as a mobility aid. A+H was the walker in Configuration (A) with a haptic user interface for signaling the navigational guidance. B+A was the walker in configuration (B) with an audio feedback. To model severe visual impairment, the users (healthy volunteers) were blindfolded.

We chose these setups to investigate some representative choices at various technology levels. Thus, BASELINE is a “no-tech”, traditional assistive device. A+H is the “mid-tech” choice, it uses a relatively simple sensor technology, with a low dimensional output (seven dimensions), simple user interface based on a binary haptic actuator. As a perceived complexity, the code driving this system can be measured in about one hundred lines of Python code, without relying on external libraries. The B+A setup is the “high-tech” choice: the input technology is the video input at a resolution of 1024 × 1024 corresponding to a dimensionality of one million. It is processed through a deep learning system combining several technologies (ResNet, Inception, FasterRCNN) with the number of parameters exceeding 10 million. It also uses a high-level, voice-based output. Naturally, many other combinations could be investigated. However, a full exploration of the possible pairings are beyond the scope of this paper.

We performed two types of experiments: simple navigation from source to destination point and more complex indoor navigation experiments. In both types of experiments, we measured the number of time the users collided with the obstacles. There were two major situations that led to hit obstacles. In the first type, the user was already moving when the notification was issued, and the momentum of the movement led to hitting the obstacle. In the second type, a notification about an obstacle led the user to change their trajectory, and this led to immediately hitting another obstacle, different from the one about which it had been notified. We conjecture that the first type of collision could be mitigated with a faster overall process of the detection–decision–notification cycle. The second type would require the system to have a more sophisticated navigation and user prediction model, which would take into account more obstacles in the scene and model the user’s likely reaction to the navigation command. We have not encountered a situation where the obstacle detector would have missed one of the obstacles in the scene.

The detection range of the HC-SR04 ultrasonic sensor is 4 m, the one of the Kinect sensor is about 3.5 m, and the one based on a camera is basically unlimited, in practice extending to the nearest occlusion. We judge that these ranges are sufficient for obstacle detection in a rollator scenario. The practical problem, however, is that it is impractical for a system to make a notification when it sees an obstacle four meters away—in any scenario there is always going to be some kind of obstacle that is far out. The main challenge, as we noted above, is not the detection of the obstacle, but making the decision to notify the user in the right way and at the right time. If we notify the user too early, we risk spamming them about obstacles they will not hit anyhow, while if we notify them too late, the user is already committed to a move and will collide with the obstacle despite the notification.

#### 4.3.1. Simple Navigation

The simple navigation task involved the user moving from a source to a destination location, on a trajectory that would be a straight line in the absence of obstacles. The experiments involved navigating an environment with various obstacle densities. We used both an indoor and an outdoor environment, with the type of obstacles suitable to the setting. The experiments considered four levels of obstacle densities: empty, low, medium and high. We used the same ten obstacles, but the higher the obstacle density, the closer the obstacles were to each other leading to a more complex navigation task. Note that even for the empty setting, the user had to avoid collision with walls for the indoor environment and curbs and cars for the outdoor environment. For each experiment, we counted the number of obstacles the user collided with during the movement, as well as the time it took to navigate from the source to the destination.

**Figure 6:**
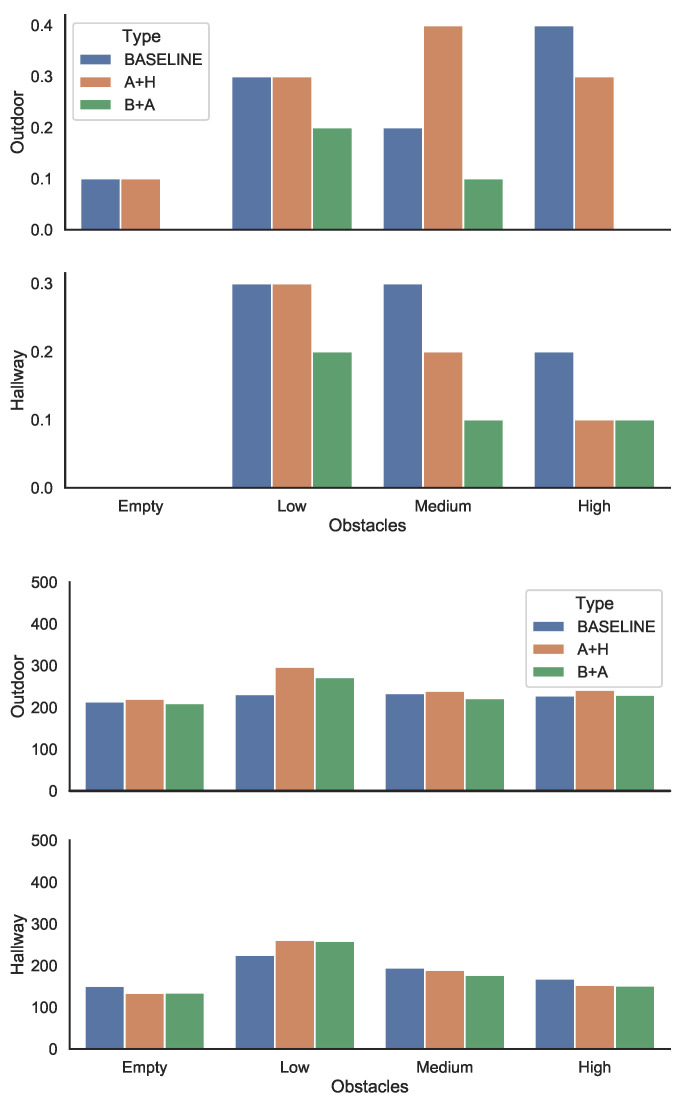
(top) Efficacy of the navigational guidance system for configurations A and B, measured as the percentage of the obstacles in the environment that were hit during the navigation (lower is better); and (bottom) time needed to perform a navigation task (lower is better).

Figure 6—top shows the percentage of the obstacles hit under various configurations, as an average of two trials. The results show that while there is considerable individual variation, overall, the more densely packed the obstacles were, the lower the number of the collisions and the faster the traversal. We conjecture that the main reason for this is that when the obstacles were closely clustered, the user could traverse the area with the obstacles very carefully and slowly to avoid them, but it could speed up on the rest of the trajectory. However, when the same number of obstacles was distributed in the whole area (in this case, with lower density) the user was more likely to be taken by surprise by an obstacle.

Comparing the three setups, the B+A approach consistently showed the lowest number of collisions. The A+H approach was in general better than the baseline, with one outlier for the medium-density outdoor setting. Empirical observation led us to conjecture that this was due to a “snowball” effect: if a user hits several obstacles in close succession, it is likely that they will hit other ones as well, perhaps as a result of disorientation.

These results validate the fact that the walkers need to be evaluated as a holistic system. While both configurations correctly detected the obstacles, with in fact the ultrasonic sensor in A+H being the more accurate, the overall results in setup B+A were better. We conjecture that the reason for this is that the users were not accustomed to navigate based on the haptic feedback, with the audio signals offering a clearer guidance. In addition, the vision-based sensing combined with audio output allowed the walker to identify the obstacle. This information was not available in setup A+H. This information could be used by the user for a more successful navigation.

This finding matches other studies that investigate the cognitive load of audio and haptic feedback in assistive systems. For instance, Martinez et al. [28] found that blind people prefer haptic feedback over audio feedback for short range navigation tasks, but prefer audio feedback for other tasks such as orientation, communication and alerts.

Figure 6—bottom shows the measured values for the time to reach the destination.

For the indoor environment, we found that the higher the density of the obstacles, that is, the closer the obstacles were clustered together, the faster the traversal time, as the trajectory contains large stretches where no obstacle was present, allowing the user to speed up. However, we found that, in general, there is little difference between the different walker configurations in the time taken to navigate the trajectory.

#### 4.3.2. Complex Indoor Navigation Task

**Figure 7:**
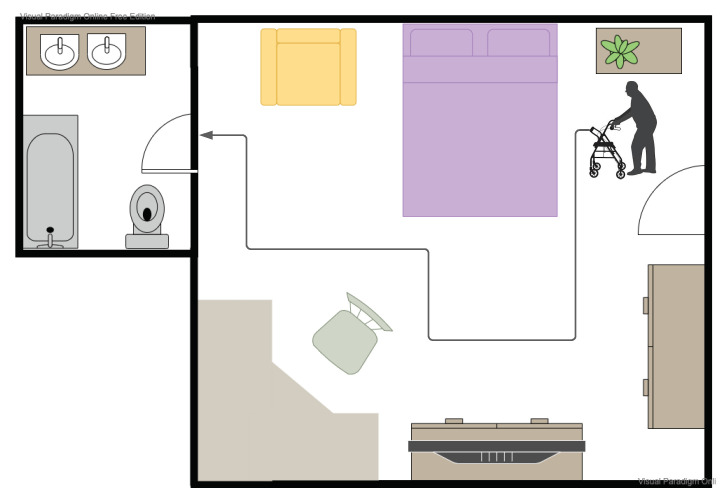
A complex navigation task in an indoor environment. The disabled user needs to navigate from the bedroom to the bathroom, avoid obstacles such as the bed and the chair, and must find and open the appropriate doors.

The most frequently encountered navigation task by a mobility and visually challenged person is navigating their personal environment: this involves moving from the bedroom to bathroom or from the front room to the kitchen. In contrast to the fixed source–destination pairs we considered in the previous section, these navigation tasks are more complex: they involve finding paths, going through doors and maneuvering around furniture (see Figure 7).

To investigate the impact of the different walker setups for this task, we measured the user’s navigation of four different paths in a house. The experiments were repeated with the BASELINE, A+H and B+A configurations. The time it took to navigate these paths and the number of obstacles with which they collided is shown in Table 3. In this environment, we found that both augmented configurations A+H and B+A allowed the user to complete the navigation tasks, both faster and with a lower number of collisions compared to the BASELINE. There was no significant difference between the two augmented configurations.

## 5. Conclusions

In this paper, we described and studied several prototypes for a smart walker specialized for people with both visual and mobility impairments. As a first conclusion, we found that there are multiple, very different choices of sensors that can ultimately ensure a similar user experience. Active sensors such as ultrasonic distance sensors or infrared depth cameras achieve the best accuracy in localizing obstacles. However, recent advances in computer vision, in particular object detection and recognition, allow passive, inexpensive cameras to achieve accuracy that is sufficient for the purposes of such a walker. In addition, computer vision systems can provide additional functionality such as identifying and naming the type of obstacle encountered by the user. Another conclusion of our experiments is that the performance of such a walker needs to be evaluated in a holistic way—the accuracy and reliability of the sensor, the type of user interaction used (such as haptic or audio), the friendliness and clarity of the user interaction and the low latency all contribute to the overall performance of the walker. Not every configuration justifies the additional cost of the technology. In particular, it is not enough that there is a sensor that detects the obstacle—we also need to find a way to convey it to the user in a way that triggers the right real-time obstacle avoidance behavior.

## Figures and Tables

**Table 1:** Comparing the accuracy of configurations A and B for measuring the distance (cm) to several indoors (upper part) and outdoors (lower part) obstacle types.

Obstacle	Ground Truth	Config (A)	Config (B)
Empty hallway	N/A	No obstacle	No obstacle
Wall	91.9	92.3	82.9
Door	53.7	54.6	40.3
Person	110.3	110.2	120.9
Stairs	51.8	52.3	54.9
Backpack	42.5	42.6	39.5
Curb	87.3	86.9	100.3
Car	132.6	131.3	111.2
Swimming pool	49.3	50.1	68.3

**Table 2:** Comparing the accuracy of configurations C and D for measuring the distance (cm) to several likely obstacle types.

Obstacle	Ground Truth	Config (C)	Config (D)
Empty hallway	N/A	No obstacle	No obstacle
Wall	128	130	120
Drop	N/A	Detected	Detected
Bottom of stairwell	125	129	119
Item in path	182	187	172

**Table 3:** Experimental results for the complex navigation tasks.

Type of Aid	Scenario	Time	Obstacles Hit
BASELINE	front door to kitchen	2:39	3
	kitchen to bedroom	1:31	4
	bedroom to bathroom	1:28	3
	patio	3:32	3
A+H	front door to kitchen	1:52	1
	kitchen to bedroom	1:12	2
	bedroom to bathroom	1:04	1
	patio	2:31	2
B+A	front door to kitchen	1:50	1
	kitchen to bedroom	1:10	1
	bedroom to bathroom	1:09	2
	patio	2:23	0

## Data Availability

Not applicable.
